# Supplementary material for: Egalitarian cooperation linked to central oxytocin levels in communal breeding house mice
Source: Commun Biol. 2024 Sep 27;7:1193. doi: 10.1038/s42003-024-06922-y (PMC11436823; doi:10.1038/s42003-024-06922-y)
Supplement: Supplementary file 1 — Supplementary Information [file 42003_2024_6922_MOESM1_ESM.pdf]

Electronic supplementary material for:

Egalitarian cooperation linked to central oxytocin levels in communal breeding house mice

Stefan Fischer †*[1,2,3], Callum Duffield †[1], William T. Swaney[4], Rhiannon L. Bolton[1], Amanda J. Davidson[1], Jane L. Hurst[1] & Paula Stockley*[1]

†These authors contributed equally

* Corresponding authors, email: p.stockley@liverpool.ac.uk; Stefan.Fischer@vetmeduni.ac.at

[1]Mammalian Behaviour & Evolution Group, Department of Evolution, Ecology and Behaviour, University of Liverpool, Leahurst Campus, Neston, CH64 7TE, UK. [2] *Current address (1)*: Konrad Lorenz Institute of Ethology, Department of Interdisciplinary Life Sciences, University of Veterinary Medicine Vienna, Savoyenstrasse 1, 1160 Vienna, Austria. [3]*Current address (2)*: Department of Behavioral & Cognitive Biology, University of Vienna, University Biology Building (UBB), Djerassiplatz 1, 1030 Vienna, Austria. [4]School of Biological and Environmental Sciences, Liverpool John Moores University, Byrom Street, Liverpool L3 3AF.

The following supplemental material contains additional methodological details (Supplementary Note 1), a summary of the number of trials and subjects within each experimental treatment across both experiments (Supplementary Table 1), additional tables (Supplementary Tables 2-12) and a figure (Supplementary Figure 1) to support the conclusions presented in the main body of the manuscript, and details of each of the statistical models presented in the results section (Supplementary Table 13).

Supplementary Note 1

Enclosures

Trials for both experiments were conducted using the same experimental enclosures in a blocked design. Each trial consisted of a social group of mice housed in one of eight melamine enclosures (120 x 120 x 80cm), within the same environmentally controlled and ventilated animal room (temperature 20–21°C, relative humidity 45–65%, and a reversed 12:12h light cycle with lights off at 08:00, with 20 room air changes per hour). Enclosures were split into four equal interconnected compartments, each containing food and water, paper wool nesting material, a PVC tunnel for enrichment, and a protected or unprotected nest box, according to the experimental design (Fig. 1). In experiment 1, enclosures contained either four protected nest boxes, or one protected and three unprotected nest boxes. Protected nest boxes consisted of opaque plastic boxes (11.8cm x 19.8cm x 9cm [W x L x H]) with ventilation holes, paper wool nesting material, a lid, and a single entrance hole. These were placed in the corner of enclosures with access via a clear plastic sheltered entrance tunnel. Non-protected nest boxes were open opaque plastic boxes (11.8cm x 11.8cm x 6cm [W x L x H]) with nesting material but no lid. In experiment 2, all enclosures contained one protected and three unprotected nest boxes (Fig. 1). In experiment 2, the subjects' territory was also connected to a neighbouring enclosure (120 x 60 x 80 cm) via four Perspex tunnels (5cm diameter), blocked with wire mesh. In both experiments, to quantify time spent with offspring by subjects, the entrance tunnel leading to their nest was automatically monitored by two portable transponder readers connected to a customized data logger (Francis Scientific Instruments, UK). Transponder readers always recorded the nest site with the litter(s) present and were switched between nest sites to follow the location of breeding females in cases where multiple protected nest sites were available (experiment 1).

Social groups

Each social group contained four adult females, consisting of two litter-mate sister pairs of contrasting age to provide a naturalistic grouping. Older sisters were given the opportunity to breed and were the subjects of both experiments. In experiment 1, the relatedness of older and younger sisters was manipulated to investigate effects of persistent competition

with non-kin in the same territory on oxytocin levels. To manipulate relatedness, sister pairs were either derived from the same parents (full siblings) or from different parents (unrelated). In experiment 2, older and younger sisters were always related (full siblings), but from different litters, and the presence or absence of two unrelated females in the neighbouring enclosure was manipulated to investigate the effects of persistent competition with neighbours on oxytocin levels. The two females in the neighbouring enclosure were sisters, and age matched to the older subjects. Younger sisters and neighbours were always unfamiliar to subjects at the start of both experiments.

Following introduction using an ethical protocol to prevent escalated aggression, groups were allowed to establish social relationships for a minimum of one week. Subjects were then given the opportunity to mate with an unrelated adult male (introduced to the enclosure for a period of 7-10 d after initial priming with male scent) and to rear their offspring within a monitored nest box. During this reproductive phase, younger females were housed within a grill-topped MB1 cage inside the enclosure to allow interaction through the grill while preventing mating and infanticide. To maintain their social odours throughout the enclosure during this phase, younger sisters were released twice weekly for 1-2 h (without access to breeding males or to the nest box of breeding females) and their cage was repositioned twice weekly. Male odour from singly housed males was also added to enclosures weekly to maintain exposure to the social cues of a territorial male, as would occur in natural populations. To simulate territory intrusions by neighbours in experiment 2, the barriers between territories were removed for 1 hour every two weeks, with subjects confined to prevent direct aggression. To expose subjects to scent marks deposited by neighbouring females in experiment 2, Perspex tiles (15x15x0.5 cm) were positioned near territory boundaries and exchanged between neighbouring enclosures weekly. These same procedures were performed for trials with no neighbours present in experiment 2.

Maternity analysis

Offspring were weaned and removed from enclosures between post-natal day 28-30. To determine maternity of offspring, DNA was extracted using a QIAGEN DNeasy Blood & Tissue Kit (QIAGEN, UK) from a 2 mm ear punch (sampled post-mortem or under anaesthesia with isoflurane (2%) in oxygen). We genotyped parents and offspring to establish haplotypes using 11 microsatellite markers (D2Mit277, D4Mit17, D4Mit139,

D4Mit164, D4Mit217, D6Mit138, D11Mit4, D14Mit132, D17Mit22, D17Mit126, D17Mit234) from Sherborne, et al. [1] and Firman and Simmons [2]. Forward primers for each marker were 5'-fluorescently labelled with 6-FAM, PET, NED or VIC. The loci were organized into four multiplex loading groups, containing mixed loci from 7 regions. PCR amplification reactions were performed in a 10µl volume of ~20 ng DNA, 10µM of each primer and 5µl of BioMix Red reaction mix (Bioline, UK). The PCR protocol steps were: an initial denaturation for 5 min at 94 °C, 35 cycles of denaturation at 95 °C for 30 s, annealing at 60 °C -56 °C for 30 s, extension at 72 °C for 30 s, and after the 35 cycles were complete a final extension at 72 °C for 5 min. The PCR reactions were then diluted to 16- to 25-fold (depending on primer set) and multiplexed in formamide with GeneScan LIZ500 size standard (Applied Biosystems). Haplotype size was determined with an Applied Biosystems Genetic Analyser 3500XL and GeneMapper v3.0 software (Applied Biosystems). To assign maternity we compared markers carried by offspring to those carried by known sires and unknown mothers.

Oxytocin assay

To quantify the concentration of oxytocin ligand within the PVN, samples were homogenised prior to oxytocin enzyme immunoassay (EIA) analysis. Microcentrifuge tubes containing PVN samples were placed on ice, and 100µl of ice-cold Radioimmunoprecipitation assay (RIPA) buffer (ThermoFisher Scientific, UK) containing a 1:100 Halt™ protease inhibitor cocktail (ThermoFisher Scientific, UK) was added before homogenising for two minutes. Samples were then incubated for 15 minutes on ice and centrifuged at 12000*xg* for 15 minutes at 4 °C, before storage at -80 °C. If a pellet formed, the supernatant was removed and stored at -80 °C [3,4].

Oxytocin was analysed using a commercially available EIA (ADI-900-153, Enzo, USA). Reagents were brought to room temperature for 30 minutes. Standards were made up following the manufacturer's protocol: a 10,000pg/ml oxytocin standard solution was serial diluted to 1000, 500, 250, 125, 62.5, 31.2 and 15.6pg/ml. Standards and samples were plated onto a goat anti-rabbit IgG microtiter plate in 100µl aliquots, in duplicate. 50µl of oxytocin conjugate (alkaline phosphatase conjugated with oxytocin) and 50µl of rabbit polyclonal antibody to oxytocin were added to each well. Plates were tapped to mix reagents, sealed and incubated at 4 °C for 18 hours. Wells were then emptied, washed three times with washing buffer, and tapped dry on lint-free paper. 5µl of blue conjugate was

added to the total activity wells and 200µl of para-Nitrophenylphosphate (pNpp) substrate was added to each well. Plates were left to incubate at room temperature for 1 hour before adding 50µl of stop solution to each well, and read immediately at 405nm on a Thermo Scientific Multiskan FC microplate photometer (ThermoFisher Scientific, UK).

Oxytocin assay validation

We conducted tests of parallelism and accuracy to validate the measurement of oxytocin in mouse PVN samples. A serial dilution of pooled PVN samples was run in duplicate alongside a serial dilution of synthetic antigen standard (in triplicate) for standard concentrations of 15.6, 31.2, 62.5, 125, 250, 500 and 1000pg/ml. Serial dilutions of pooled PVN samples resulted in a displacement curve parallel to that of the standard curve (no difference in the slopes following linear regression, F = 0.006, P = 0.94, n =7). To conduct a matrix interference assessment, serial dilutions of synthetic oxytocin standard (125, 250, 500 and 1000 pg/ml) were spiked with equal volumes of sample at a working dilution of 1:8. No significant interference was found, as confirmed by linear regression analysis (R^2 = 0.99, F = 203, p = 0.005, n=4). Average recovery was 93.4%.

Normalising oxytocin to protein concentration within PVN homogenate samples

Prior to quantifying protein concentration accurately, an approximate concentration was estimated to facilitate production of appropriate dilutions for the EIA. 1µl samples were pipetted onto nitrocellulose paper alongside protein (bovine serum albumin) standards (1mg/ml and 2mg/ml), and left to dry for 5 minutes before Ponceau S stain was added, followed by a further 5 minute incubation period. The stain was then washed off thoroughly with ddH_20, and protein content was determined by visual comparison with the standards. Protein concentration in PVN homogenates was then quantified using a Coomassie plus® protein assay reagent kit (Perbio Science, UK) [5]. Firstly, PVN homogenates were diluted 1:200 with ddH_20, then 100µl of sample was pipetted in duplicate into a 96 well plate (Sterilin Microplate F Well 611F96, Thermo Fisher, Finland), and standards of bovine serum albumin (concentration range 0-50µg/ml) prepared from a stock solution of 2mg/ml were added to the same plate. Following this, 200µl of Coomassie reagent was added to each well and the absorbance was read at 620nm using a Thermo Scientific Multiskan FC microplate

photometer (ThermoFisher Scientific, UK). The protein concentration for each sample was calculated by interpolation from the standard curve.

Supplementary Table 1. Summary of the number of subjects and trials for which data on PVN oxytocin concentration were available. Subjects were from two experiments with independent manipulation of access to protected nest sites and outgroup competition (see Fig. 1 and Methods for further details). An initial maximum sample size of 32 subjects (16 sister dyads) was available for experiment 1 and 40 subjects (20 sister dyads) for experiment 2. The maximum available sample size was subsequently reduced to 24 for experiment 1, as PVN samples were not collected for two trials (four subjects), and the EIA analysis for four subjects did not meet quality control criteria. Columns show sample sizes utilised in key analyses (see Results and Supplementary Table 13 for further details). Sums within each column show the sample sizes from each experiment, and totals show the maximum sample sizes available for analysis across both experiments. Data on PVN oxytocin concentrations were available for a total of 64 subjects from 34 trials. Oxytocin data were available for both subjects in 30 trials and for one subject in four trials. Maternity of weaned offspring could not be reliably assigned for all offspring in two of 30 trials with oxytocin data for both subjects. In one case, we were able to determine that both subjects had produced weaned offspring, but could not determine the number of offspring produced by each subject. This dyad was therefore included in analyses of communal breeding where data on the individual reproductive success of subjects was not a factor. In the second case we were unable to determine if both subjects had produced weaned offspring. This dyad was therefore unavailable for analyses of communal or single breeding, and for analyses of individual reproductive success. There was also a single trial in which oxytocin data were available for both subjects but neither produced weaned offspring. Overall, therefore, we were able to confirm that offspring were reared in a communal nest in 15 trials and in a single nest in 13 trials. Maximum available sample sizes were used for all analyses, with the exception that two trials were excluded from analyses of reproductive success and reproductive skew: one with illness-related late-stage offspring mortality (for which maternity was only partially allocated), and one where neither subject produced weaned offspring (because we could not exclude that failure to breed was due to the male used in this trial). Overall, we were able to quantify the total number of weaned offspring produced by 43 subjects that had oxytocin data and bred successfully, excluding the trial with late-stage mortality of offspring.

**Experiment**	**Protected nest sites**	**Outgroup competition (competition with unrelated females)**	**Total number of subjects/trials with oxytocin data**	**Number of trials with oxytocin data for both subjects where offspring were reared in communal/single nests**	**Number of subjects with oxytocin data that bred successfully, and for which offspring numbers were reliably quantified**
**1**	Multiple	Yes (same territory)	7/4	1/2	5
	Multiple	No	5/3	1/1	4
	Single	Yes (same territory)	6/4	1/0	4
	Single	No	6/3	1/2	4
**Sum**			24/14	4/5	17
**2**	Single	Yes (neighbouring territory)	20/10	3/3	15
	Single	No	20/10	8/5	11
**Sum**			40/20	11/8	26
**Total**			**64/34**	**15/13**	**43**

Supplementary Table 2. Individual PVN oxytocin concentrations were significantly correlated within sister dyads. Subjects were from two experiments with independent manipulation of access to protected nest sites and outgroup competition. Shown are results from a linear mixed model. To analyse the relationship within sister dyads sharing the same enclosure, we used the PVN oxytocin concentration of the female with the higher value as the dependent variable, and of the female with the lower value as the independent variable. Experimental treatments (protected nest site availability and outgroup competition), subjects´ age, average body mass and experiment (1 or 2) did not influence this relationship and were dropped from the final model. To obtain p-values an F-test was used to compare models with and without the factor of interest. To obtain normally distributed residuals all continuous variables were log transformed. N=30 sister dyads in 10 blocks and two experiments (Supplementary Table 1).

**Factors**	**Estimate ± SE**	**Num. D.F.**	**Den. D.F**	**F-value**	***P*-value**
Intercept	3.0 ± 0.81	-	-	-	-
Individual PVN oxytocin	0.6 ± 0.13	1	24.79	21.16	**<0.01**

Supplementary Table 3. Body mass and age did not predict individual PVN oxytocin concentrations. Subjects were from two experiments with independent manipulation of access to protected nest sites and outgroup competition. Results are shown from a linear mixed model. To obtain p-values an F-test was used to compare models with and without the factor of interest. To obtain normally distributed residuals the dependent variable was log transformed. N=63 females in 34 trials across 10 blocks and two experiments (Supplementary Table 1). One subject could not be included in this analysis because no body mass measurement was available.

**Factors**	**Estimate ± SE**	**Num. D.F.**	**Den. D.F**	**F-value**	***P*-value**
Intercept	6.72 ± 0.58	-	-	-	-
Body mass (g)	-0.01 ± 0.03	1	57.21	0.19	0.66
Age (days)	$1e^{-4} \pm 1e^{-3}$	1	7.3	0.01	0.9

Supplementary Table 4. Individual PVN oxytocin concentrations did not differ according to whether subjects produced weaned offspring or not. Subjects were from two experiments with independent manipulation of access to protected nest sites and outgroup competition. Results are shown from a linear mixed model. The estimate is shown as difference to the reference level ‘no’ for the factor ‘Whether subject produced weaned offspring’. Experiment (1 or 2) and whether a communal nest was formed (yes or no) did not influence PVN oxytocin concentration of subjects and were removed from the final model. To obtain p-values an F-test was used to compare models with and without the factor of interest. To obtain normally distributed residuals the dependent variable was log transformed. N=60 females in 32 trials across 10 blocks and two experiments (Supplementary Table 1).

**Factors**	**Estimate ± SE**	**Num. D.F.**	**Den. D.F**	**F-value**	***P*-value**
Intercept	6.49 ± 0.13	-	-	-	-
Whether subject produced weaned offspring (yes, no)	0.03 ± 0.15	1	47.12	0.03	0.85

Supplementary Table 5: The duration of time between removal of weaned offspring and collection of PVN samples did not predict individual PVN oxytocin concentrations. Subjects were breeding females from two experiments with independent manipulation of access to protected nest sites and outgroup competition. Results are shown from a linear mixed model. To obtain p-values an F-test was used to compare models with and without the factor of interest. To obtain normally distributed residuals all continuous variables were log transformed. N=46 females in 10 blocks and 2 experiments (Supplementary Table 1).

Factors	Estimate ± SE	Num. D.F.	Den. D.F	F-value	*P*-value
Intercept	-0.18 ± 0.24	-	-	-	-
Number of days	-0.01 ± 0.01	1	24.57	1.08	0.31

Supplementary Table 6. **Average PVN oxytocin concentrations of sister dyads did not predict communal breeding.** Subjects were from two experiments with independent manipulation of access to protected nest sites and outgroup competition. Results are shown from a generalised linear mixed model with a logit link. Experimental treatments (protected nest site availability and outgroup competition), subjects' age, average body mass and experiment (1 or 2) did not influence communal breeding and were dropped from the final model. To obtain p-values a likelihood-ratio test (χ^2-test) was used to compare models with and without the factor of interest. N=28 sister dyads across 10 blocks and two experiments (Supplementary Table 1).

**Factors**	**Estimate ± SE**	**X^2-value**	***P*-value**
Intercept	-0.77 ± 1.15	-	-
Average PVN oxytocin (pg/mg protein)	$1e^{-3} \pm 1e^{-3}$	0.72	0.39

Supplementary Table 7. Factors predicting the number of weaned offspring produced by individual dams. Subjects were from two experiments, with independent manipulation of access to protected nest sites and outgroup competition. Results are shown from a linear mixed model. Experimental treatments (protected nest site availability and outgroup competition), maternal age, maternal body mass and experiment (1 or 2) did not influence weaned offspring numbers and were dropped from the final model. Estimates are shown as difference to the reference level 'no' for the factor 'Communal nest' (whether a communal nest was formed). To obtain normally distributed residuals the dependent variable was log transformed. To obtain p-values an F-test was used to compare models with and without the factor of interest. N=43 females in 30 trials in 10 blocks across two experiments (Supplementary Table 1).

**Factors**	**Estimate ± SE**	**Num. D.F.**	**Den. D.F**	**F-value**	***P*-value**
Intercept	1.17 ± 1	-	-	-	-
Individual PVN oxytocin concentration (pg/mg protein)	0.02 ± 0.15	1	40	0.01	0.91
Communal nest (yes/no)	0.4 ± 0.17	1	40	5.49	**0.02**

Supplementary Table 8. **Factors predicting reproductive skew between sister dyads cooperating in a communal nest.** Subjects were from two experiments with independent manipulation of access to protected nest sites and outgroup competition. Results are shown from a linear mixed model. Reproductive skew is analysed as the difference in the number of weaned offspring produced between subjects sharing a communal nest, controlling for the combined number of weaned offspring produced. Due to the low sample size, we did not include experimental treatments (protected nest site availability and outgroup competition) or experiment (1 or 2) in the full model. To obtain p-values an F-test was used to compare models with and without the factor of interest. N=13 sister dyads across eight blocks and two experiments (Supplementary Table 1).

**Factors**	**Estimate ± SE**	**Num. D.F.**	**Den. D.F**	**F-value**	***P*-value**
Intercept	0.3 ± 2.31	-	-	-	-
Average PVN oxytocin (pg/mg protein)	$-4e^{-3} \pm 2e^{-3}$	1	10	5.42	**0.04**
Combined number of weaned offspring	0.49 ± 0.25	1	10	3.88	*0.08*

Supplementary Table 9. Relationship between time spent in the nest and time spent feeding by subjects that produced a litter. Subjects were from experiment 2 with independent manipulation of the presence or absence of neighbours. Results are shown from a linear mixed model. Experimental treatment (presence or absence of neighbours) and whether a communal nest was formed (yes/no) did not influence time spent in the nest and were dropped from the final model. To obtain normally distributed residuals the dependent variable was log transformed. To obtain p-values an F-test was used to compare models with and without the factor of interest. N=21 females in 13 trials across five blocks (see Methods for further explanation of this behavioural assay).

**Factors**	**Estimate ± SE**	**Num. D.F.**	**Den. D.F**	**F-value**	***P*-value**
Intercept	7.49 ± 0.71	-	-	-	
Time spent feeding	-0.27 ± 0.09	1	18.5	9.61	**0.01**

Supplementary Table 10. Factors predicting relative time spent in the nest by sister dyads cooperating in a communal nest during the inactive (light) period. Relative time spent in the nest was calculated as the difference in the proportion of total time spent in the nest by each subject. Results are shown from a linear mixed model. Subjects were from two experiments with independent manipulation of access to protected nest sites and outgroup competition. The availability of protected nest sites (multiple, single) and experiment (1 or 2) did not influence the relative time spent in the nest and were dropped from the final model. Estimates are shown as differences to the reference level 'no' for factor 'Outgroup competition'. To obtain p-values an F-test was used to compare models with and without the factor of interest. To investigate the significant main effect 'Outgroup competition' we performed pairwise comparisons of the three levels ('no', 'yes [same territory]' and 'yes [neighbouring territory']) with a Tukey correction for multiple testing and results are presented in Supplementary Table 11. N=15 sister dyads across eight blocks and two experiments (Supplementary Table 1).

**Factors**	**Estimate ± SE**	**Num. D.F.**	**Den. D.F**	**F-value**	***P*-value**
Intercept	0.04 ± 0.04	-	-	-	-
Average PVN oxytocin (pg/mg protein)	$-1e^{-5} \pm 4e^{-5}$	1	8	0.16	0.7
Outgroup competition (*yes[same territory], yes [neighbouring territory], no*)	-	2	10.12	6.43	**0.02**
Outgroup competition (yes[same territory])	*7e-3 ± 0.05*				
Outgroup competition (yes[neighbouring territory])	*0.1 ± 0.03*				

Supplementary Table 11: The influence of outgroup competitors on the relative time spent in the nest by sister dyads cooperating in a communal nest during the inactive (light) period. Pairwise comparisons of the three-level factor ‘Outgroup competition’ with a Tukey correction for multiple testing are presented. The comparisons were done after confirming that the main effect ‘Outgroup competition’ was significant in Supplementary Table 10. The direction of comparison within a contrast is left to right and the estimate value represents the difference to the level on the left. Cooperating sister dyads spend more similar amounts of time in the nest during the light (inactive) phase when outgroup competitors were absent compared to when outgroup competitors were present in the neighbouring territory but not when outgroup competitors were present in the same territory. N=15 sister dyads across eight blocks and two experiments (Supplementary Table 1)

**Contrast**	**Estimate ± SE**	**Num. D.F.**	**Den. D.F**	**t-value**	***P*-value**
‘no’ vs. ‘yes[same territory]’	-0.01 ± 0.04	1	8.24	-0.16	0.97
‘no’ vs. ‘yes[neighbouring territory]’	-0.1 ± 0.03	1	10.64	-2.84	**0.04**
‘yes[same territory]’ vs. ‘yes[neighbouring territory]’	-0.1 ± 0.04	1	7.75	-2.06	0.16

Supplementary Table 12: Individual PVN oxytocin concentrations were unrelated to absolute time spent in the nest with pups during the (a) active (dark) period or (b) inactive (light) period. Subjects were from two experiments with independent manipulation of access to protected nest sites and outgroup competition. Results are shown from two linear mixed models. Experimental treatments (protected nest site availability and outgroup competition), experiment (1 or 2), and whether the subject gave birth did not influence the absolute time spent in the nest with pups and were dropped from the final model. To obtain p-values an F-test was used to compare models with and without the factor of interest. N=56 females in 30 trials across 10 blocks and two experiments (Supplementary Table 1).

Factors	Estimate ± SE	Num. D.F.	Den. D.F	F-value	*P*-value
**(a) Absolute time spent in the nest with pups during the active (dark) period**					
Intercept	$2.7e^{5} \pm 3.2e^{4}$	-	-	-	-
Individual PVN oxytocin concentration (pg/mg protein)	$2.3e^{4} \pm 3.6e^{4}$	1	50.86	0.43	0.52
**(b) Absolute time spent in the nest with pups during the inactive (light) period**					
Intercept	$3.9e^{5} \pm 3.4e^{4}$	-	-	-	-
Individual PVN oxytocin concentration (pg/mg protein)	$-1.1e^{3} \pm 3.6e^{4}$	1	48.94	$1e-^{3}$	0.98

Supplementary Table 13. Information on the linear mixed models (LMM) and a generalized linear mixed model (GLMM) analysed during this study, including the respective response variables, explanatory variables, covariates and random factors, eventual data transformations performed to obtain normally distributed residuals, and any interaction terms included in the initial, full models. If data included zeros and log transformations had to be applied, we added a constant value to all data points (i.e. 1, or in cases with data points below 1, the value closest to 0). To obtain final models, non-significant covariates and interactions were stepwise removed. Explanations of response variables: 'Individual PVN OT': PVN oxytocin concentration for an individual subject; 'Combined nr. of weaned offspring': the combined number of weaned offspring produced by a sister dyad within a given trial; 'Relative time spent in the communal nest': the difference in the proportion of total time spent in the communal nest by each subject, separated into active (dark) and inactive (light) periods; 'Higher PVN OT': Subjects within each trial were classified as having a higher or lower PVN oxytocin concentration relative to one another; 'Whether a communal nest was formed': whether a communal nest was formed (yes/no) in a given trial; 'Nr. of weaned offspring': the number of weaned offspring produced by individual dams. 'Reproductive skew': the difference in the number of pups weaned by communal breeding subjects; 'Time spent in the nest': the absolute time spent in the nest during an assay conducted in the active (dark) phase for subjects that weaned a litter in experiment 2. 'Time in nest during the active (dark) period': the absolute time spent in the nest from post-natal day 0-14 during the active (dark) period; 'Time in nest during the inactive (light) period': the absolute time spent in the nest from post-natal day 0-14 during the inactive (light) period. Explanatory variable names: 'Protected nest sites': whether multiple or single protected nest sites were available; 'Outgroup competition': whether subjects experienced long-term exposure to social cues of outgroup competitors (unrelated females), and whether outgroup competitors were within the same territory or neighbours in an adjacent territory; 'Average PVN OT': average PVN oxytocin concentration of the two subjects in the same trial; 'Lower PVN OT': the lower PVN oxytocin concentration recorded for subjects within each sister dyad; 'Body mass': the body mass of subjects; 'Age': the age of subjects; 'Whether subject produced weaned offspring': whether a subject produced weaned offspring (yes/no) in a given trial; 'Nr. of days until sample': number of days between the day that weaned offspring

were removed and the day of the brain sample collection; ‘Time spent feeding’: the absolute time spent feeding during an assay for subjects that weaned a litter in experiment 2. Covariate names: ‘Experiment’: whether data was obtained in experiment 1 or 2; ‘Average body mass’: average body mass of the two subjects in the same trial; ‘Combined nr. of weaned offspring’: the combined number of weaned offspring produced by a sister dyad within a given trial. Random factor names: ‘Sister dyad ID’: identification number of sister dyads sharing the same nest or enclosure; ‘Block ID’: identification number of blocks 1-10.

**Response variable**	**Explanatory variables**	**Covariates**	**Random factor**	**Transformation or Link**	**Interactions**	**Final model**
Individual PVN OT	Protected nest sites<br>Outgroup competition	Age<br>Body mass<br>Experiment	Sister dyad ID<br>Block ID	Log<br>(LMM)	None	Table 1
Combined nr. of weaned offspring	Average PVN OT	Whether a communal nest was formed (yes/no)<br>Experiment<br>Protected nest sites<br>Outgroup competition<br>Age<br>Average body mass	Block ID	None<br>(LMM)	Average PVN OT x Whether a communal nest was formed	Table 2a
Combined nr. of weaned offspring	Average PVN OT	None	Block ID	None<br>(LMM)	None	Table 2b
Combined nr. of weaned offspring	Average PVN OT	None	Block ID	None<br>(LMM)	None	Table 2c
Relative time spent in the communal nest during the dark period	Average PVN OT	Experiment<br>Protected nest sites<br>Outgroup competition	Block ID	None<br>(LMM)	None	Table 3

Higher PVN OT	Lower PVN OT	Experiment<br>Age<br>Average body mass<br>Protected nest sites<br>Outgroup competition	Block ID	Log<br>(LMM)	None	Supplementary Table 2
Individual PVN OT	Body mass<br>Age	None	Sister dyad ID<br>Block ID	Log<br>(LMM)	None	Supplementary Table 3
Individual PVN OT	Whether subject produced weaned offspring (yes/no)	Experiment<br>Whether a communal nest was formed (yes/no)	Sister dyad ID<br>Block ID	Log<br>(LMM)	None	Supplementary Table 4
Individual PVN OT	Nr. of days until sample	None	Sister dyad ID<br>Block ID	Log<br>(LMM)	None	Supplementary Table 5
Whether a communal nest was formed (yes/no)	Average PVN OT	Experiment<br>Protected nest sites<br>Outgroup competition<br>Age<br>Average body mass	Block ID	Logit link<br>(GLMM)	None	Supplementary Table 6
Nr. of weaned offspring	Individual PVN OT	Age<br>Body mass<br>Whether a communal nest was formed (yes/no)<br>Experiment<br>Protected nest sites<br>Outgroup competition	Sister dyad ID<br>Block ID	Log<br>(LMM)	Individual PVN OT x Whether a communal nest was formed	Supplementary Table 7
Reproductive skew	Average PVN OT	Combined nr. of weaned offspring	Block ID	None<br>(LMM)	None	Supplementary Table 8

Time spent in the nest	Time spent feeding	Whether a communal nest was formed (yes/no)<br>Outgroup competition	Sister dyad ID<br>Block ID	Log<br>(LMM)	None	Supplementary Table 9
Relative time spent in the communal nest during the inactive (light) period	Average PVN OT	Experiment<br>Protected nest sites<br>Outgroup competition	Block ID	None<br>(LMM)	None	Supplementary Table 10
Time in nest during the active (dark) period	Individual PVN OT	Whether subject produced weaned offspring (yes/no)<br>Protected nest sites<br>Outgroup competition<br>Experiment	Sister dyad ID<br>Block ID	None<br>(LMM)	None	Supplementary Table 12a
Time in nest during the inactive (light) period	Individual PVN OT	Whether subject produced weaned offspring (yes/no)<br>Protected nest sites<br>Outgroup competition<br>Experiment	Sister dyad ID<br>Block ID	None<br>(LMM)	None	Supplementary Table 12b

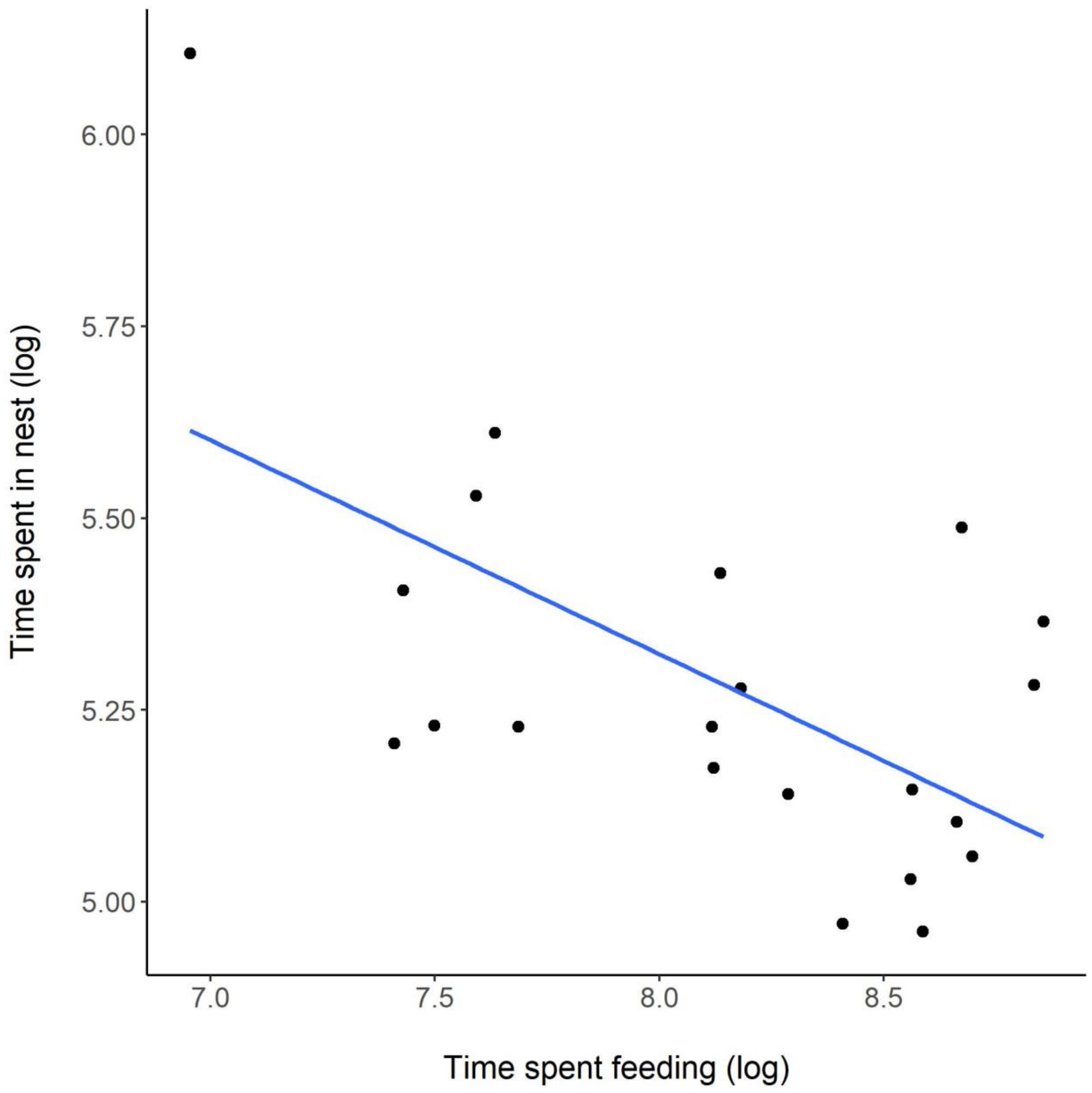

Supplementary Figure 1. **Relationship between time spent in the nest and time spent feeding for subjects that weaned a litter.** Subjects were from experiment 2 with independent manipulation of the presence or absence of neighbours. Time spent in the nest was recorded continuously from birth to postnatal day 14, and time spent feeding was assayed daily between post-natal day 10-14 during the dark (active) period. Overall time spent in the nest was negatively correlated with time spent feeding. This relationship was not influenced by experimental treatment (presence or absence of neighbours) or whether or not subjects raised their litter in a communal nest. See Supplementary Table 9 for statistical analysis.

Supplementary References

1 Sherborne, A. L. *et al.* The genetic basis of inbreeding avoidance in house mice. *Curr. Biol.* **17**, 2061-2066 (2007).

2 Firman, R. C. & Simmons, L. W. The frequency of multiple paternity predicts variation in testes size among island populations of house mice. *J. Evol. Biol.* **21**, 1524-1533 (2008).

3 Tait, S. *et al.* Long-term effects on hypothalamic neuropeptides after developmental exposure to chlorpyrifos in mice. *Environ. Health Persp.* **117**, 112-116 (2009).

4 Zhang, H. F. *et al.* Electro-acupuncture improves the social interaction behavior of rats. *Physiol Behav* **151**, 485-493 (2015).

5 Cheetham, S. A. *et al.* The genetic basis of individual-recognition signals in the mouse. *Current Biology* **17**, 1771-1777 (2007).
