# Supplementary material for: Egalitarian cooperation linked to central oxytocin levels in communal breeding house mice
Source: Commun Biol. 2024 Sep 27;7:1193. doi: 10.1038/s42003-024-06922-y (PMC11436823; doi:10.1038/s42003-024-06922-y)
Supplement: Supplementary file 2 — Reporting Summary [file 42003_2024_6922_MOESM2_ESM.pdf]

nature portfolio

Corresponding author(s): Paula Stockley

Last updated by author(s): Sep 5, 2024

Reporting Summary

Nature Portfolio wishes to improve the reproducibility of the work that we publish. This form provides structure for consistency and transparency in reporting. For further information on Nature Portfolio policies, see our Editorial Policies and the Editorial Policy Checklist.

Statistics

For all statistical analyses, confirm that the following items are present in the figure legend, table legend, main text, or Methods section.

n/a	Confirmed	
☐	☒	The exact sample size (*n*) for each experimental group/condition, given as a discrete number and unit of measurement
☐	☒	A statement on whether measurements were taken from distinct samples or whether the same sample was measured repeatedly
☐	☒	The statistical test(s) used AND whether they are one- or two-sided<br>*Only common tests should be described solely by name; describe more complex techniques in the Methods section.*
☐	☒	A description of all covariates tested
☐	☒	A description of any assumptions or corrections, such as tests of normality and adjustment for multiple comparisons
☐	☒	A full description of the statistical parameters including central tendency (e.g. means) or other basic estimates (e.g. regression coefficient) AND variation (e.g. standard deviation) or associated estimates of uncertainty (e.g. confidence intervals)
☐	☒	For null hypothesis testing, the test statistic (e.g. *F*, *t*, *r*) with confidence intervals, effect sizes, degrees of freedom and *P* value noted<br>*Give P values as exact values whenever suitable.*
☒	☐	For Bayesian analysis, information on the choice of priors and Markov chain Monte Carlo settings
☒	☐	For hierarchical and complex designs, identification of the appropriate level for tests and full reporting of outcomes
☐	☒	Estimates of effect sizes (e.g. Cohen's *d*, Pearson's *r*), indicating how they were calculated

Our web collection on statistics for biologists contains articles on many of the points above.

Software and code

Policy information about availability of computer code

Data collection	RFID logging equipment and software from Francis Scientific Instruments were used to monitor time subjects spent in the nest.
Data analysis	Data analyses were performed in R (v 4.1.1) (cited reference 77). The R-code used to generate all models and figures are provided as Supplementary information deposited in the Figshare repository.

For manuscripts utilizing custom algorithms or software that are central to the research but not yet described in published literature, software must be made available to editors and reviewers. We strongly encourage code deposition in a community repository (e.g. GitHub). See the Nature Portfolio guidelines for submitting code & software for further information.

Data

Policy information about availability of data

All manuscripts must include a data availability statement. This statement should provide the following information, where applicable:

- Accession codes, unique identifiers, or web links for publicly available datasets
- A description of any restrictions on data availability
- For clinical datasets or third party data, please ensure that the statement adheres to our policy

The datasets analysed during the current study are provided as Supplementary information deposited in the Figshare repository

Research involving human participants, their data, or biological material

Policy information about studies with human participants or human data. See also policy information about sex, gender (identity/presentation), and sexual orientation and race, ethnicity and racism.

Reporting on sex and gender	NA
Reporting on race, ethnicity, or other socially relevant groupings	NA
Population characteristics	NA
Recruitment	NA
Ethics oversight	NA

Note that full information on the approval of the study protocol must also be provided in the manuscript.

Field-specific reporting

Please select the one below that is the best fit for your research. If you are not sure, read the appropriate sections before making your selection.

☐ Life sciences ☐ Behavioural & social sciences ☒ Ecological, evolutionary & environmental sciences

For a reference copy of the document with all sections, see nature.com/documents/nr-reporting-summary-flat.pdf

Ecological, evolutionary & environmental sciences study design

All studies must disclose on these points even when the disclosure is negative.

Study description	We conducted two experiments to investigate if the cooperative behaviour and reproductive success of female house mice is related to variation in their oxytocin production, and if oxytocin levels are influenced by: i) limited availability of protected nest sites for breeding, or ii) long-term exposure to social cues of outgroup competitors, either within the same territory or as neighbours in an adjacent territory. Oxytocin concentrations in the paraventricular nucleus (PVN) of the hypothalamus were quantified at the end of each experiment to test for predicted relationships between PVN oxytocin levels and reproductive success, and for differences in PVN oxytocin levels explained by environmental conditions. Both experiments were conducted in enclosures under the same controlled conditions, providing subjects with the opportunity to breed communally within a social group. Social groups each contained four adult females, consisting of two litter-mate sister pairs of contrasting age to provide a naturalistic grouping. Older sisters were given the opportunity to breed and were the subjects of both experiments (experiment 1: n= 32; experiment 2: n = 40). In experiment 1, the social environment was manipulated by varying the availability of protected nest sites for breeding, and the presence or absence of unrelated competitors living within the subjects' territory. In experiment 2, competition between social groups was manipulated by varying the presence or absence of unrelated competitors living in a neighbouring territory. To determine if subjects bred communally, we combined behavioural observations of nest sharing with maternity analysis of weaned offspring to confirm that both sisters had contributed to a communal litter. For sisters sharing offspring care, we also quantified the relative time each spent in the communal nest as a measure of cooperative behaviour. To quantify time spent with offspring by communally breeding females, the entrance tunnel leading to their nest was automatically monitored. To test if the relative time spent in the nest by communal breeding females was influenced by their average PVN oxytocin concentrations, we calculated the difference in the proportion of total time spent in the nest by each sister. We also assayed time spent feeding for a subset of subjects, to confirm that time spent in the nest by cooperating females is a useful indirect measure of their relative investment in the communal litter. At the end of both experiments, all subjects were culled humanely and brain micro-dissections were carried out to obtain tissue samples from the PVN. Oxytocin was analysed in PVN homogenate using a commercially available EIA (ADI-900-153, Enzo, USA) following manufacturer's instructions. PVN homogenate oxytocin concentrations were successfully quantified for 64 subjects (n = 24 in experiment 1 and n= 40 in experiment 2) from 34 trials. To determine maternity of weaned offspring, we genotyped parents and offspring to establish haplotypes. To assign maternity we compared markers carried by offspring to those carried by known sires and unknown mother(s). Reproductive success was quantified as the number of weaned offspring produced. At least one subject within a sister dyad produced weaned offspring in 33 trials, and maternity was assigned unambiguously for 252 of 256 weaned offspring produced by subjects with known PVN oxytocin concentrations. This resulted in full maternity allocation of weaned offspring for 28 sister dyads, including 15 cases where a communal nest was formed and 13 where no communal nest was formed and only one sister produced weaned offspring.
Research sample	Subjects were outbred wild-stock female house mice derived from ancestors captured from six populations in the northwest of England, UK, mated to unrelated males from the same stock population. The regular addition of new wild-caught animals to this captive population ensured that subjects reflected the normal genetic variation of wild house mice, and had not undergone prolonged artificial selection in the laboratory.
Sampling strategy	Sample sizes are based on previous studies with wild mice, including pilot data and consideration of published effect sizes using similar approaches where available. To provide a naturalistic environment for wild house mice, the experiments were conducted on subjects living in small social groups. Social groups each contained four adult females, consisting of two litter-mate sister pairs of contrasting age. Subjects were the older sister dyad within each group, and were given the opportunity to breed with an unrelated

male. Subjects (sister dyads) were randomly allocated to experimental treatments. In experiment 1, the relatedness of older and younger sisters in the same social group was manipulated to investigate effects of outgroup competition on oxytocin levels. To manipulate relatedness, sister pairs were either derived from the same parents (full siblings) or from different parents (unrelated). In experiment 2, older and younger sisters were always related (full siblings), and the presence or absence of two unrelated females in the neighbouring enclosure was manipulated to investigate the effects of outgroup competition with neighbours on oxytocin levels. The two females in the neighbouring enclosure were sisters, and age matched to the older subjects. Younger sisters and unrelated females were always unfamiliar to subjects at the start of both experiments. Subjects were given the opportunity to mate with an unrelated adult male and to rear their offspring within a monitored nest box. Trials in both experiments were run in blocks. To control for potential differences between experimental blocks, we always included 'block ID' as a random effect in analyses. Average values of PVN oxytocin concentrations and other traits such as combined number of weaned offspring were calculated for sister dyads within each trial. If individual data for both subjects from the same trial were used, we included 'sister dyad ID' as a random effect. To analyse whether the presence of unrelated competitors influenced dependent variables we used a factor 'Outgroup competition' with three levels to test for an overall effect of the presence of outgroup competition across both experiments, and for separate effects of outgroup competition within the same social group or with a neighbouring group. To analyse whether the number of protected nest sites available influenced dependent variables we used a factor 'Protected nest sites' with two levels: multiple or single protected nest sites available. We checked for any remaining variation explained by differences between experiments using the factor 'experiment' with two levels (experiment 1 or 2). However, this factor was not a significant predictor of the dependent variable in any of the analyses and was dropped from the final models.

Data collection	Both experiments were run in the Mammalian Behaviour & Evolution laboratories at the University of Liverpool by SF (experiment 1) and CD (experiment 2), who set up the trials, monitored subjects' behaviour and breeding success, downloaded RFID monitoring data from data loggers, carried out brain micro-dissections (with help from WS), giving PVN samples unique sample numbers, and ran oxytocin assays (with help from RB and AJD). Additional assays of feeding behaviour were conducted during experiment 2 by CD. Tissue samples for maternity analysis were each given unique sample numbers; DNA was extracted by AJD who carried out microsatellite genotyping in the Centre for Genomic Research at the University of Liverpool. RFID files were sent to JLH and CD for calculation of the amount of time that communal breeding females spent with pups in the nest box. All data were then collated by CD and SF.
Timing and spatial scale	Experiments began in January 2016 and were completed (including maternity analysis of weaned offspring) by February 2022. Spatial scale is not relevant to this study.
Data exclusions	Maximum available sample sizes were used for all analyses, with the exception that two trials were excluded from analyses of reproductive success and reproductive skew: one with illness-related late-stage offspring mortality (for which maternity was only partially allocated), and one where neither subject produced weaned offspring (because we could not exclude that failure to breed was due to the male used in this trial). Details are provided in Supplementary Table 1.
Reproducibility	Two experiments were run across a total of 34 trials for which reliable measures of PVN oxytocin were obtained from at least one subject, with data overall for 64 subjects. The number of subjects and trials available for each analysis are detailed in the methods and supplementary information (Supplementary Table 1).
Randomization	Treatment groups were approximately balanced across blocks, with sister dyads randomly allocated to treatment groups within this restriction.
Blinding	Data on time spent in the communal nest were collected automatically. Samples for PVN oxytocin EIAs and genotyping to determine reproductive success were given unique sample numbers, and assays and molecular analysis were conducted blind to treatment group.

Did the study involve field work? ☐ Yes ☒ No

Reporting for specific materials, systems and methods

We require information from authors about some types of materials, experimental systems and methods used in many studies. Here, indicate whether each material, system or method listed is relevant to your study. If you are not sure if a list item applies to your research, read the appropriate section before selecting a response.

Materials & experimental systems

n/a	Involved in the study
☒	☐ Antibodies
☒	☐ Eukaryotic cell lines
☒	☐ Palaeontology and archaeology
☐	☒ Animals and other organisms
☒	☐ Clinical data
☒	☐ Dual use research of concern
☒	☐ Plants

Methods

n/a	Involved in the study
☒	☐ ChIP-seq
☒	☐ Flow cytometry
☒	☐ MRI-based neuroimaging

Animals and other research organisms

Policy information about studies involving animals; ARRIVE guidelines recommended for reporting animal research, and Sex and Gender in Research

Laboratory animals	Animals used in the study were captive-bred adult wild-stock house mice (Mus musculus domesticus) and their offspring.
Wild animals	The study did not involve wild (free-living) animals.
Reporting on sex	The study is designed to focus on female behaviour. Sex-based analyses were not required
Field-collected samples	The study did not involve samples collected in the field.
Ethics oversight	All animal care protocols were in accordance with the University of Liverpool Animal Welfare Committee requirements and UK Home Office guidelines for animal care. Tissue samples from live animals were obtained under UK Home Office licence according to best practice and guidelines and approved by the University of Liverpool Animal Welfare Committee.

Note that full information on the approval of the study protocol must also be provided in the manuscript.
